# Perceptual breakdown during a global pandemic: introducing phenomenological insights for digital mental health purposes

**DOI:** 10.1007/s10676-020-09554-y

**Published:** 2020-09-01

**Authors:** Janna van Grunsven

**Affiliations:** Technische Universiteit Delft, South Holland, Delft, The Netherlands

**Keywords:** Digital mental health, Loss of perceptual world-familiarity, COVID-19 pandemic, Phenomenology, Well-being, Affordances, Ego-depletion, Online therapy, Social interaction technologies

## Abstract

Online therapy sessions and other forms of digital mental health services (DMH) have seen a sharp spike in new users since the start of the COVID-19 pandemic. Having little access to their social networks and support systems, people have had to turn to digital tools and spaces to cope with their experiences of anxiety and loss. With no clear end to the pandemic in sight, many of us are likely to remain reliant upon DMH for the foreseeable future. As such, it is important to articulate some of the specific ways in which the pandemic is affecting our self and world-relation, such that we can identify how DMH services are best able to accommodate some of the newly emerging needs of their users. In this paper I will identify a specific type of loss brought about by the COVID-19 pandemic and present it as an important concept for DMH. I refer to this loss as *loss of perceptual world-familiarity.* Loss of perceptual world-familiarity entails a breakdown in the ongoing effortless responsiveness to our perceptual environment that characterizes much of our everyday lives. To cash this out I will turn to insights from the phenomenological tradition. Initially, my project is descriptive. I aim to bring out how loss of perceptual world-familiarity is a distinctive form of loss that is deeply pervasive yet easily overlooked—hence the relevance of explicating it for DMH purposes. But I will also venture into the space of the normative, offering some reasons for seeing perceptual world-familiarity as a component of well-being. I conclude the paper with a discussion of how loss of perceptual world-familiarity affects the therapeutic setting now that most if not all therapeutic interactions have transitioned to online spaces and I explore the potential to augment these spaces with social interaction technologies. Throughout, my discussion aims to do justice to the reality that perceptual world-familiarity is not an evenly distributed phenomenon, that factors like disability, gender and race affect its robustness, and that this ought to be reckoned with when seeking to incorporate the phenomenon into or mitigate it through DMH services.

## The perceptual world as a place of familiarity^1^

Many of the forms of loss people have experienced as a result of the COVID-19 pandemic have been nothing short of devastating. Under dehumanizing conditions of isolation, people have lost loved ones along with the traditional rituals for grieving them, people have lost their jobs, homes, trust in government, access to education (and, for some children, the safe-haven school provides from distressing if not dangerous home-situations). People who have contracted COVID-19 themselves and ended up in the ICU will have temporarily lost the ability to breathe independently (for a staggering 10 days on average). Those who survive intubation and wake up from their induced coma may struggle with the long-term loss of various physical and cognitive abilities.^2^

But even if one has had the fortune to remain free from these palpable forms of loss, I believe the pandemic has also enabled the experience of a less visceral but nevertheless pervasive form of loss, which I refer to as *loss of perceptual world-familiarity*. To explain this, let me specify what notion of *perceptual world* I am working with. Following the phenomenological tradition and insights from the field of embodied cognition, I understand the perceptual world as a space of *affordances*. The notion of affordances, coined by ecological psychologist J.J. Gibson, connotes the idea that living beings perceive their environment in terms of the practical possibilities for action it affords them (Gibson, 1979; see also Rietveld and Kiverstein 2014; Dreyfus 2007). What organizes a living being’s perceptual world as a space of affordances are first and foremost its needs, skills and habits as an embodied being. When I am hungry and have the skill to handle knives, I typically directly perceive my freshly purchased groceries as affording to-be-eaten and my kitchen knife as affording to-be-picked up. When I am in a rush to get home and have the ability to close doors and press buttons, I directly perceive the front door to my apartment building as affording to-be-opened and the elevator button as affording to-be-pushed. Our social lives are equally saturated with affordance-guided interactions. When we have the capacity for empathy and we share a background understanding of what grieving together involves, we may directly perceive the trembling body of another person as affording to-be-comforted in context appropriate ways. When we are trained as a psychologist and have an established history of interactions with a client, we likely directly perceive the psychological significance of that client’s non-verbal bodily expressions as affording a particular form a response. The perceptual world as a space of affordances is “present as a *familiar* setting of our life” (2012, 53). It is a place where we typically feel at home.

What the above examples illustrate is that for the embodied situated living agent there is an internal link between how things are perceived and how they are responded to, enabling situation-specific ways of acting intelligently in the world without the involvement of thematic reflective thought. When I walk into a crowded subway car I typically do not have to thematize the number of passengers around me to take up an appropriate distance from them, nor do I have to pay attention to the shape of the subway pole in order to be able to grab it and maintain my balance. I effortlessly and habitually negotiate these features of my social and practical environment, I dwell pre-reflectively in this practical setting while focusing thematically on, say, a conversation I am having with a friend. In fact, my ability to remain focused on the conversation *depends* upon my situatedness in an environment upon which I do not need to reflect in order to know my way about it. This claim about the dependency of our thematic world-directedness on our background familiarity with the perceptual world as a space of affordances is in part a claim about our ability to *mentally off-load.* If I had to focus on how to shape my hand in order to grip the subway pole appropriately, while also calculating deliberately if I had managed to obtain an appropriate distance from the other passengers on the train, I would simply never get to the conversation I might hope to have with my friend. It is because the perceptual world as a space of affordances tends to recede into the background while we cope with it skillfully yet pre-reflectively, that we can ‘get on with our lives’ and focus our attention and effort on what needs careful deliberation, planning or thought.

Though pre-reflective and nearly automatic, it should be clear by now that these ways of engaging the world are far from thoughtless; they exhibit a kind of practical know-how—a way of knowing one’s way about in the world that reflects a nuanced contextual form of understanding. Martin Heidegger’s favorite example to illustrate this point is that of a hammer being used to hit a nail into a piece of wood. In handling a hammer ‘mindlessly’ so to speak, one is manifesting a rich understanding of not just the hammer but of the entire use-context that gives the hammer as a tool its specific practical sense—we understand our way around hammers, nails, work-benches and exhibit an understanding of what they are used for. We understand what Heidegger terms a “totality of equipment,” or what I will call a *micro-world* (Heidegger 1962). In Sect. 4, I will approach the therapeutic setting as a micro-world, asking how the COVID-19 pandemic and the relocation of in-person therapy to online spaces has altered the affordances characteristic of this micro-world.

Sometimes we experience a breakdown in one of the many micro-worlds we inhabit: a sudden flat tire disrupts my biking and makes me examine my tire *as* a distinct object, a misplaced hammer that I anticipated to be within reach makes me look for it as a thing I am missing. When this happens, a certain distance is created between that micro-world and myself: I am no longer absorbed in the flow of my activity, I stand over and against the world, encountering myself as someone whose ongoing coping has been frustrated. In the words of Charles Taylor: “I may begin to classify things as ‘obstacles’ or ‘facilitations,’ and this will change the way I live in the world” (2005, 34)*.* This image of perceptual breakdown and the transformation it brings about in our self and world-relation offers a helpful lens for looking at some of the effects of the COVID-19 pandemic. As I will now suggest, SARS-CoV-2 and the measures adopted to help flatten the curve have, in numerous ways, disrupted the perceptual world as a place of familiarity.

## COVID-19 and the breakdown of perceptual world-familiarity

Though much about the SARS-CoV-2 virus is still shrouded in uncertainty, there seems to be a growing consensus among virologists and epidemiologists that the virus can spread asymptomatically. Furthermore, it has been shown that the virus can remain contagious on certain surfaces, such as plastic, for several days.^3^ Airborne transmissions of the virus also seems to occur (Cf. Wilson et al 2020). Finally, though we know age, comorbidity, profession, sex, and race make a statistically relevant difference, it is ultimately (still) unpredictable why some people succumb to the virus and why others don’t. These facts about the virus make nearly every feature of our environment a potential source of infection and every person a potential threat to our health and livelihood. Many of the most basic features of our practical environment (door-handles, elevators, public transportation, cash, produce, our mail) seem to warrant a new form of engagement (I have a relative who irons the newspaper before handing it to her health-compromised husband. In a similar vein countless people are treating their produce with cleaning agents, sometimes with disastrous consequences).^4^ Even our bodily self-relation has not remained unscathed as many of us monitor not just our hands touching the everyday objects around us but our very own nose, mouth, eyes (ironically, the roughness of my hands from the continual hand-washing conveniently alerts me during moments of forgetting). The transformation of social affordances—both in the private sphere and the public domain—have been even more dramatic. Strangers on the street largely afford to-be-shunned or avoided. At home, when we’ve fallen ill, our typical ways of seeing and responding to each other as affording to-be-cared-for have taken on unrecognizable shapes as well. There may be no comforting hands of a loved-one on one’s forehead gauging one’s temperature, no arms helping one get up out of bed, no cups of comforting tea put on your bedside table. One is likely left alone in a room with a thermometer, seeing mere traces of the other—that cup of tea now left in front of one’s bedroom door. Thus, while social distancing is medically mandated, it is profoundly disrupting the fabric of our social affordances and the interactional flow often characteristic of face-to-face responsiveness.

We are dealing, then, not just with a particular moment of breakdown of a particular micro-world, momentarily throwing us back onto ourselves, disrupting our activity—we are experiencing moments of breakdown in nearly every region of our everyday world of affordances. Of course, the degree to which one is sensitive to the new affordances COVID-19 has introduced will vary at cultural and individual levels. But even if one hasn’t incorporated the ‘to-be-feared’ and ‘to-be-avoided’ as new affordances in one’s world—even if one isn’t ironing newspapers, disinfecting door-handles, drenching one’s groceries in (sometimes hazardous) cleaning agents, or keeping physical distance from a coughing loved one at home, the societal measures implemented to flatten the curve are nudging (nearly) everyone to relate to public space and its (social) affordances differently; many of us no longer dwell in public spaces the way we used to—we no longer casually grab the subway pole and rely on our habitual know-how to take up an appropriate distance to others. Moving about in public space is now more often than not an effortful endeavor.

I have been suggesting that a new cluster of affordances, the ‘to-be-avoided,’ the ‘to-be-suspected’, the ‘to-be-treated-with-caution’ has been layered over many of the everyday objects and people we interact with in our everyday lives. It isn’t exactly right to say that our old affordances have disappeared altogether. Of course, we also still perceive keys and door handles as affording-to-be-grasped; the apple we just purchased as affording-to-be-eaten mindlessly; the strangers we see on the elevator as affording-to-be-greeted, our sick loved ones as affording to-be-held and cared for. The key thing to notice, though, is that the current situation invites us to *simultaneously* see these objects and persons as affording to be feared, avoided, treated with caution, where this new affordance is in direct conflict with the old affordances we still perceive as well. There is a fundamental incompatibility between the ‘to-be-feared’ or ‘to-be-avoided’ that is now tethered to many of our everyday use-objects and social encounters and the effortlessness with which we used to respond to them. We can find an analogue to this kind of experience in Merleau-Ponty’s account of the phantom limb:

To have a phantom limb is to remain open to all of the actions of which the arm alone is capable and to stay within the practical field that one had prior to the mutilation. … But at the very moment that the world hides [the patient’s] … deficiency from him, the world cannot help but to reveal it to him. … *At the same moment that my usual world gives rise to habitual intentions in me, I can no longer actually unite with it* (2012, 84, my italics).

This strikes me as the right kind of imagery for capturing the phenomenology of our current situation. We still see the world in terms of its old familiarity, but it is a familiarity that is at the same time alienating as we are no longer able to respond to its call in the habitual effortless manner characteristic of the pre-pandemic world we inhabited. If there is indeed an experiential similarity between our current failure to “unite with” our “usual world” and the experience of world-loss recounted by persons with an acquired disability, there seems to be an opportunity here to learn from and to deepening our empathy with disabled people and to promote a societal acknowledgment of the ways in which all of our lives as agents depend intimately on the degree to which we can inhabit a world that affords uniting with. Though it is beyond the scope of this paper to discuss these suggestive remarks in detail, I do revisit the importance of acknowledging the perspectives of differently situated and differently embodied persons when it comes to theorizing the notion of perceptual world-familiarity and incorporating it into DMH services.

## Perceptual Breakdown and well-being

So far, I have discussed the notion of perceptual world familiarity in largely descriptive terms. But I think what we are simultaneously seeing in between the lines, is that it carries normative significance. Arguably, perceptual world-familiarity can be seen as a component of well-being understood in the Eudaimonian sense of living well, of thriving in one’s activities of self-actualization. One way to bring this out more explicitly is as follows: when illustrating the phenomenon of unreflective effortless responsiveness to perceptual affordances, Hubert Dreyfus tends to invoke images from the world of professional sports (Cf. 2007). Athletes but also dancers and musicians on top of their game exhibit levels of elegance and effortlessness in how they move about in their micro-world that tend to evoke images of freedom, vitality, excellence. If the notion of thriving, of actualizing one’s human abilities well, don’t apply to these instances of human activity it arguably never does. It would be a gross exaggeration to say that we are thriving in this pregnant sense when we mindlessly store away our groceries, effortlessly manipulate door handles, automatically take up an appropriate distance from others on the train or at the local supermarket.^5^ However, it does seem right to say that a continual breakdown in a wide array of our perceptual affordances and effortless responsiveness to them will have a significant impact on one’s well-being. If the unreflective activities that tend to support our more labor-intensive thematic forms of world-directedness now warrant thematic directedness themselves, this creates the condition for a specific kind of fatigue stemming from excessive self-monitoring and of reorienting oneself in a world that has lost some of its immediate action-guiding significance. Flow-like engagements are continuously interrupted by attitudes of distrust towards and detachment from the familiar.

To put this in terms familiar to psychologists, the loss of perceptual world-familiarity brought about by the pandemic can be understood as a distinct source of *ego-depletion*.^6^ Ego-depletion refers to the (temporary) loss of subjective vitality or the “feeling of aliveness and energy” (Ryan and Frederick 1997, 529), resulting from strenuous efforts of exercising self-control, where self-control is understood as “a deliberative, conscious, effortful, and resource intensive process of restraining an impulse in order to reach a long-term goal or follow a rule” (Muraven 2012, 111). Mark Muraven discusses the pervasive effects of ego depletion on a person’s overall well-being. Invoking an abundance of empirical research, he maintains that ego-depletion affects not only one’s ability to control (undesirable) impulses, but also one’s basic cognitive processes, one’s experience of time, one’s willingness to initiate new action and overcome passivity, and one’s responsiveness to societal norms of social interaction (2012). This suggests that loss of world-familiarity and the “conscious, effortful” self-monitoring and environment-directedness stemming from it can have a clear impact on well-being.

Note that perceptual world-familiarity understood as a dimension of well-being works in a similar vein as *trust*—we experience its normative significance, the central role it plays in our daily functioning and vitality, precisely when it has been disrupted—when we can no longer take it for granted as the pervasive supporting background to our goals and activities as agents (Cf. Baier 1986).^7^ And just like trust, the robustness of perceptual world-familiarity—the degree to which it manages to stay in the background, quietly supporting one’s daily functioning—is not distributed evenly. The phenomenological image of the embodied skillful agent effortlessly at home in a world of perceptual affordances has been the subject of a feminist line of critique. Iris Marion Young, for instance, accuses Merleau-Ponty of implicitly assuming the perspective of a male able-bodied perspective onto the world when he characterizes the lived body as the locus of effortless openness onto the world (1980). One privileged perspective is universalized and what is ignored or downplayed is that this kind of world-relation is not available to everybody equally. A parking garage or a quiet street after dark will likely afford a different kind of engagement for a woman—one much less characterized by unreflective absorption—than for a man. To the extent that we see this as *unfair* or *unjust*, though, I would argue that this precisely underscores the normative significance of perceptual world-familiarity for well-being. Put differently, presumably the different material, technological and institutional changes we can bring about in the world that could minimize the ways in which women, but also persons of color, members of the LGBTQ community, or persons with disabilities disproportionately experience breakdown in their perceptual world-familiarity supportive of their daily functioning, are changes *for the better*. In the next section, this thought operates in the background as I discuss the challenges but also the possibilities laid bare by the COVID-19 pandemic and its resultant shift from therapy conducted in-person to therapy conducted online.

## Digital Mental health and the loss of perceptual world-familiarity

What relevance does the phenomenon of loss of perceptual world-familiarity have for DMH? Firstly, a general remark that applies not just to *digital* mental health services but to mental health services more broadly. If my analysis has been right, then loss of perceptual world-familiarity is a pervasive yet easily overlooked phenomenon plausibly experienced on a widespread global scale as a result of the pandemic. Understanding this phenomenon (and making it visible as a distinct source of fatigue or ego-depletion) could help prevent large-scale mental health misdiagnoses and misdirected forms of treatment, thus potentially alleviating an already overburdened mental health system. Experiences of sudden large loss require space for grief (rather than, say, anti-depressants)^8^. That grief could indeed be seen as an appropriate response to perceptual world-loss can be motivated via a characterization of grief offered by Michael Westerlund, who in turn invokes psychiatrist and phenomenologist Karl Jaspers’s notion of a *limit situation*. Westerlund suggests that grief “should not primarily be regarded as a pathological condition…, but rather as a psychosocial and existential limit situation triggered by an overwhelming and disruptive loss in life” (2020). I wager that “a disruptive loss in life” placing us in “a psychosocial and existential limit situation” is an accurate characterization of the ubiquitous breakdown in perceptual affordances caused by the pandemic. Of course, the grief at issue here is curious in the sense that it lacks a distinct unique target—a specific someone who can be mourned. What we are grieving instead is a way of being-at-home in the world, a familiarity that is inscribed in non-codifiable forms of knowing-how to respond to affordances. Quite possibly, then, loss of perceptual world-familiarity and our grieving it take on the form of inchoate feelings, a sense that something is off, a sadness that we cannot necessarily locate or identify as easily as traditional forms of grief. A first step towards making the inchoate recognizable (and with that more manageable) is to give it conceptual articulation. And though perceptual world-familiarity as it manifests in our experiential lives seems to resist full codification, the general idea *that* the perceptual world is a place of familiarity that we can lose our grip on can by itself play a therapeutic role, providing the tools for what is sometimes called *Psychoeducation* (Bakker et al. 2016).

Not only does perceptual world-familiarity and its significance for sustaining our agential lives resist full *codification*, I also argued, at the end of the previous section, that it manifests itself in a person’s life in a manner that resists *universalization*. Each of us possesses different skills and habits, occupies different roles, identifies with different gender-norms, relies on a different set of sense-modalities, and all of this is co-constitutive of the different micro-worlds we enact and inhabit and the degree of robustness to breakdown that those worlds possess (consider, for instance, the heightened precariousness of a deaf-blind person’s perceptual world-familiarity, now that social distancing measures have made touch-based ways enacting and sustaining grip on one’s social world essentially off-limits).^9^ Thus, instead of concluding with an attempt to gleam general implications for DMH from the phenomenon of (loss of) perceptual world-familiarity, what I will do instead is home in on one micro-world in particular, namely that of the therapeutic setting itself. I will ask how the loss of world-familiarity resulting from the pandemic manifests itself in this particular micro-world: How are old affordances breaking down but, also, how are new affordances opened up? And how could *social interaction technologies* help mitigate some of this breakdown but also open up potentially significant new, digitally mediated, affordances?

### Breakdown and possibility in the micro-world of online therapy

The therapeutic setting in its traditional sense has a number of characteristic features: it is a micro-world characterized by intimate face-to-face interactions that are marked by an asymmetry in a (often) dyadic relationship. While the role of the client is to open herself up to the therapist, the therapist’s role is to deepen her understanding of the client’s lived perspective. In that sense, the work of the therapist can be described as the work of empathy. As phenomenologists have long underscored, empathy turns on a responsiveness to social affordances, particularly to the other’s subtle non-verbal bodily expressions (Cf. Stein 1989). The levels of the therapist’s responsiveness to the affordances of the other’s expressive body importantly shape the quality of the relational domain enacted through face-to-face dyadic interactions. As Feijt et al. (2018) put it: “Every response of the client, either verbal or non-verbal, is a cue conveying information to the therapist that facilitates … attunement and allows her to respond more empathically” (26).

It is undeniable that the therapeutic setting as described here has been profoundly affected by the pandemic. In a recent qualitative study involving 51 mental health practitioners from the Netherlands, Feijt et al. observe:

Whereas before the COVID-19 outbreak, online mental health tools were used infrequently, with more than half of practitioners never or hardly ever using such tools, after the outbreak this situation has changed dramatically, with the large majority of practitioners using such tools on a daily basis. This drastic change, forced by necessity, brings numerous challenges of online mental healthcare to the fore (2020).

Some of these challenges have to do with clients lacking the technical skills or the technology itself needed to even enter the online therapy space.^10^ But even with these in place, the online setting clearly disrupts many of the perceptual affordances that characterize the traditional micro-world enacted in face-to-face therapy. For starters, the other is only partially visible, such that relevant moments in posture-taking (“hand-movements, but also general demeanor”) will be hidden from view and “smell”, an important non-verbal clue, is altogether absent (Feijt et al. 2020). Furthermore, there are various sources for breakdown in the interactional flow: bad internet connections disrupt the rhythm of the interaction, direct eye-contact is nearly impossible on standard online video communication platforms such as *Zoom* or *Skype*, and the typical ways in which we might encourage, reassure, or comfort another during an interaction (reaching out with a hand, say) are unavailable. Bierbooms et al. report that many therapists see this as one of the most prominent worries about therapy conducted online:

[I]t is very difficult to connect at an interpersonal level during an online interaction, such as videoconferencing. Non-verbal cues can be easily missed, which requires a lot of additional questions and explicit feedback moments from a therapist. Moreover, participants stated multiple times that it can be very difficult to react adequately to clients’ emotions in an online therapeutic setting. … *“for example when someone starts to cry, I find it difficult to react when I am not in the same room. There is literally a distance” [P9]*] (Pre-print).

Furthermore, Bierbooms et al. note that “Multiple participants also reported to find the videoconference sessions more intensive and tiring compared to face to face sessions. *"It takes more effort to concentrate, and I am much more exhausted after a day of videocall sessions" [P7]* One way to explain this is precisely in terms of the breakdown in social affordances that typically play such an important role in establishing an empathic understanding of the other’s lived perspective. Essential features of the therapy session that used to unfold nearly habitually now require “a lot of additional questions and explicit feedback.” Without mitigating these issues, we could face a situation in which the therapists we depend on now more than ever experience a form of ego-depletion. Furthermore, we could face a situation in which the loss of world-familiarity that the client may already encounter in her day-to-day life off-line is exacerbated in the online environment precisely meant to help her cope with such loss.

Feijt et al. (2018) propose that some of the issues raised above can be mitigated by embedding social interaction technologies in the online platforms used for real-time interpersonal therapy. They note that in adopting this approach, there are essentially two different visions, the *traditional* and the *complementary* approach. The traditional approach, they explain,

is to support or simulate face-to-face interactions as closely as possible, thereby attempting to overcome limitations imposed by the medium (e.g., supporting eye contact or gesturing). Developing and applying technologies to meet a basic level of empathic interaction is in line with this approach (26).

While it is important to explore how the breakdown of affordances characteristic of the traditional micro-world of face-to-face therapy can be mitigated by attempting to simulate that world using technological functionalities such as eye-contact and gesture supporting technologies, it should also be noted that the transition to online spaces has enacted new affordances that may, for some purposes and for some users, carry unexpected advantages.^11^ As Feijt et.al. note: “Some clients seem to benefit from the distance created by the online treatment; some become less inhibited in their expressions, while others appear to become less dependent on their therapist. *"For some clients self-disclosure is easier when they are in their home environment" [P44]* (2020). For those who find traveling daunting or who experience direct face-to-face social interaction not so much as a ground for connectedness but as a source of distress, the turn to online spaces may be a welcome one worth retaining after social distancing measures have evaporated. To echo Young’s earlier critique of Merleau-Ponty, the forced transition to online therapy may have also disclosed that the traditional micro-world of the therapeutic setting—a world marked by an ongoing intimate, in-person responsiveness to the affordances of the other’s expressive body—is a world more familiar and hospitable to some than to others. With that in mind, I suggest that the pandemic could mark a pivotal moment in the development of DMH services—a moment in which one should not rely on implicit assumptions about what ‘the’ micro-world of the therapy setting should look like such that it can be best simulated at the digital level. Though simulation will indeed be important for many users, others, for instance many persons on the autism spectrum, might benefit from a very differently designed micro-world with a different array of affordances.

That the transition to digital spaces can enact new affordances, new possibilities for action and interaction not previously available, and that this could provide us with an opportunity when it comes to DMH development is also the assumption of the second approach to embedding social interaction technologies in DMH, namely the *complementary approach*. The complementary approach embraces the altered character of the online therapeutic environment, aiming to “use unique affordances of the medium to *transform* the interaction into something that could add value above and beyond what would be possible in an unmediated encounter (Feijt et al. 2018, 26–7). In pursuing the development of these new interaction affordances, it is important to take note of the fact that the digital spaces currently supporting most of our social interaction (therapeutic or otherwise) are still largely video and audio-centric.^12^ But different modalities for promoting social interaction are being explored. Consider, for instance, some of the work done on technologically mediated bio-signal communication, where technology is used to augment digitally mediated inter-personal communication with vital markers such as pulse or heartbeat. Janssen et al. (2010) have shown that *cardiac communications*, in which we hear the other person’s heart-beat while communicating with them in a virtual space, can promote a sense of closeness and affective access to the other in a manner comparable to the ways in which mutual gaze and interpersonal distance can affect one’s sense of closeness to the other (2010). Though their research focuses on cardiac communication in the auditory modality, they add that it lends itself to haptic technologies as well, opening up potentially promising avenues for promoting more inclusive online spaces that offer meaningful affordances to those who communicate and seek ways of establishing closeness to others primarily via touch.

### A final note on collective perceptual world-loss as an opportunity for ethical reflection

Though the pandemic-induced loss of world-familiarity of which I have been speaking manifests itself differently in each of our lives, there is, at the same time, an undeniable uniqueness to the fact that this loss bears on the world *en masse* at this very moment. And though physical distancing prevents us from sharing this with others in person at a large scale, digital environments can facilitate shared ways of articulating what this loss means to us. This may also present us with an important opportunity. Earlier, I cited Westerlund, who used Karl Jasper’s notion of a limit situation to characterize grief. According to Jaspers, limit situations—though often anxiety inducing—are also situations of opportunity; situations “in which the human mind confronts the restrictions … of its existing forms … to enter new realm[s] of self-consciousness … or more reflected modes of knowledge” (Thornhill and Miron 2020). Indeed, perceptual world-familiarity as a habitual stratum of human life, though arguably essential for individual well-being, is also the locus of much in our lives as agents that warrants critical examination from an ethical standpoint. Not in the least, it warrants scrutinizing the ease with which—at a societal level—we have accepted or hardly taken notice of the fact that the world of perceptual familiarity—and its constitutive role in living well—has not been as readily accessible to all of us as it should be. Maybe it is under these alienating circumstances, in digital spaces, that we can find a middle ground between mindless habitual response and alienating detachment and breakdown; maybe it is here that we can articulate which affordances are actually worth grieving while reimagining which new affordances are worth enacting—in digital spaces and elsewhere.

## Footnotes

1 I want to thank an anonymous reviewer for their thoughtful feedback and for encouraging me to expand the final portion of this paper and asking the editor to give me additional space for doing so. I also want to thank Wijnand Ijsselsteijn, whose expertise in digital well-being and human–computer interaction I greatly benefitted from in re-writing this paper. Finally, I want to thank Erik Rietveld, with whom I have discussed many of the ideas that show up in the first half of this paper for over a decade and who provided generous feedback on the first version of this paper.

2 Cf.Dhurv Khular’s “The Challenges of Post-Covid-19 Care” (2020).

3 Cf. https://www.cdc.gov/coronavirus/2019-ncov/prevent-getting-sick/how-covid-spreads.html

4 Cf. https://www.businessinsider.nl/coronavirus-people-are-accidentally-poisoning-themselves-with-bleach-2020-4?international=true&r=US

5 Nor would it be right to see any form of detachment from our situatedness in our perceptual world of familiarity as fundamental threats to our well-being. Though some phenomenologists, Dreyfus and Sean Kelly for instance, do sometimes seem to hold this view On the contrary, detachment is necessary for many of the endeavors that give our life meaning and that can be central to well-being; scientific, aesthetic, and ethical achievements wouldn’t be possible without it (after all, these endeavors typically require – at least in part – a critical reflective distance from ourselves and the habitual flow-like engagements with our perceptual environment I have been focused on).

6 I want to thank Wijnand Ijsselsteijn for introducing me to this concept and its link to my notion of loss of perceptual world-familiarity.

7 For a powerful case-study that brings out the idea that human flourishing or well-being depends on perceptual world-familiarity see Oliver Sack’s piece on Virgil (1995).

8 I want to thank Erik Rietveld and Geerteke van Lierop for making this point explicitly, in commenting on an earlier draft.

9 https://www.newyorker.com/news/our-columnists/who-is-worthy-deaf-blind-people-fear-that-doctors-wont-save-them-from-the-coronavirus.

10 As bierbooms et al. note: Specifically, vulnerable mental health clients may lack the digital skills, cognitive ability, motivation, and/or resources to partake successfully in online digital treatment and are at risk to severely be disconnected from the care they need [e.g. 7,9].

11 In addition to embedding eye-contact and gesturing supporting technologies, Feijt at all propose that the distance between therapist and client described above may also be bridged using VR technology: “VR also offers the possibility to create shared virtual environments (VEs). Although it has not been investigated yet, one can imagine that when a therapist and client are communicating remotely but are together in the same VE, this increases mutual feelings of closeness” (Feijt et. al. 2018, 28). This is an undeniably interesting possibility, particularly during pandemic times as it might also be considered as a resource for persons to ‘meet’ a loved one to whom they can no longer say good bye in person. However, the concerns of inequity raised earlier with respect to technology-access and skill-possession, are even more likely to apply in this context than they already were in the context of the hardware and software needed for accessing commonplace online spaces.

12 I want to thank Matthew Dennis for drawing my attention to this when he shared his insights on digital well-being at the recent Webinar “Digital Technologies for Well-Being and Mental Health,” put on by the TU Delft Safety and Security Institute (https://www.tudelft.nl/en/tu-delft-safety-security-institute/events/webinars/digital-technologies-for-well-being-and-mental-health/
